# Development of a High-Throughput Indirect Competitive Chemiluminescence Enzyme-Linked Immunoassay for the Rapid Detection of Bongkrekic Acid in Tremella Fungus and Rice Noodles

**DOI:** 10.3390/foods15101749

**Published:** 2026-05-15

**Authors:** Xingdong Yang, Chenchen Wang, Lihua Wu, Yutong Cao, Yinuo Zhu, Keshi Ma, Zhonghua Liu, Xiaofei Hu

**Affiliations:** 1 College of Life Sciences and Agronomy, Zhoukou Normal University, Zhoukou 466001, China; 20151010@zknu.edu.cn (X.Y.);; 2 Key Laboratory for Animal Immunology of the Ministry of Agriculture, Henan Academy of Agricultural Sciences, Zhengzhou 450002, China

**Keywords:** bongkrekic acid, monoclonal antibody, chemiluminescent enzyme-linked immunoassay, food

## Abstract

Bongkrekic acid (BKA) is a potent respiratory toxin produced by *Pseudomonas cocovenenans*. This toxin is commonly found in spoiled fermented rice- and wheat-based products, snow fungus, and black fungus and can cause severe foodborne illness. The development of a rapid onsite detection method can effectively prevent food poisoning incidents and ensure food safety. In this study, a highly specific anti-BKA monoclonal antibody was prepared, the reaction conditions were optimized, and an indirect competitive chemiluminescent enzyme-linked immunoassay (ic-CLEIA) system was developed for high-throughput screening of BKA in food. The results showed that the ic-CLEIA had good linearity in the range of 7.3–106.6 pg/mL, a limit of detection of 4.7 pg/mL, a limit of quantification of 7.3 pg/mL, a half-maximal inhibition concentration of 28.2 pg/mL, a spike recovery of 86.6–94.1%, a coefficient of variation of less than 10%, and no cross-reactivity with structural analogs. There was no significant difference between the detection results obtained with ic-CLEIA and ultraperformance liquid chromatography–tandem mass spectrometry for the samples. This method provides reliable technical support for food safety monitoring, especially for grassroots laboratories and large-scale sample screening.

## 1. Introduction

Bongkrekic acid (BKA, C_28_H_38_O_7_) is a foodborne toxin produced by the *Pseudomonas cocovenenans* subsp. *farinofermentans* and *P. cocovenenans* subsp. *cocovenenans* [1,2]. The main target of BKA is mitochondria. It can inhibit the activity of adenine nucleoside translocase (ANT) in mitochondria, hindering the exchange of adenosine diphosphate (ADP) and adenosine triphosphate (ATP) in the inner membrane of mitochondria; this disruption poses severe obstacles to the energy supply in cells [3,4]. Owing to the stability of its molecular structure, BKA is resistant to high temperatures and difficult to degrade. For example, this substance is difficult to remove effectively through conventional food processing methods such as heating (boiling at 100 °C and high-pressure steaming at 120 °C), washing, and freezing [5]. BKA is usually found in fermented rice- and wheat-based products, spoiled snow fungus and black fungus, traditional fermented foods, and decayed potato-based or starch-rich foods [6,7]. This toxin is colorless, tasteless, and odorless, making it difficult to detect and greatly increasing the risk of food poisoning [8]. BKA is highly toxic, and humans can be poisoned and die if they ingest 1~1.5 mg BKA toxin/kg body weight. According to animal experiments, the oral median lethal dose of BKA for mice is 0.7~6.8 mg/kg body weight [9,10]. When a human ingests food containing high concentrations of BKA, the compound causes serious damage to the cells of important organs, including the liver, kidneys, and brain, producing poisoning symptoms that can even be life-threatening [11,12].

BKA poisoning is most common worldwide in Java, Indonesia; Northeast and South China; Southeast Asia; and Southern Africa [13,14,15,16]. At present, although the European Union and the United States have not set specific limit standards for BKA, they strictly regulate microbial toxins in food to ensure food safety and protect public health. China has clearly stipulated that the maximum residue limit (MRL) of BKA in Tremella and its products is 250.0 μg/kg. However, there are no corresponding standards or specifications for the MRL of BKA in other foods. China’s current “Food Safety Standard Determination of BKA in Food” (GB 5009.189-2023, [17]) uses two detection methods [high-performance liquid chromatography (HPLC) and HPLC-tandem mass spectrometry (HPLC–MS/MS)] to detect the residues of BKA in Tremella, other fungi, fermented rice noodles and their products. The limit of detections (LODs) for these two methods are 5.0 and 1.0 μg/kg, and the quantitative limits are 15.0 and 3.0 μg/kg, respectively. To prevent the recurrence of poisoning incidents and to provide a rapid diagnosis after poisoning, it is crucial to establish a rapid and sensitive detection method for BKA.

At present, various detection methods have been used for BKA analysis; the main methods are high-performance liquid chromatography (HPLC) [18], HPLC-Orbitrap HRMS [19], HPLC-diode array detection (HPLC-DAD) [20], HPLC-MS/MS [21,22], and ultraperformance liquid chromatography–MS/MS (UPLC-MS/MS) [23,24,25,26]. Although these methods are accurate and reliable, they are slow and costly processes that require expensive chromatographic equipment, complex instrument operation, complicated sample pretreatment, professional technicians, and highly controlled environments [27]. They are often used as confirmation methods. Several immunoassay methods have been reported for the detection of bongkrekic acid, such as enzyme-linked immunosorbent assays (ELISAs) [28], immunochromatographic strips [29,30], and immunosensors [31,32] (Table 1). Immunoassays, which have the advantages of being rapid, sensitive, and accurate, can meet the needs of rapid onsite screening and are widely used in food safety testing, medical inspection and environmental monitoring [33]. In particular, chemiluminescence enzyme-linked immunoassays (CLEIAs) have the merits of high sensitivity and accurate quantitative analysis and are widely used in fast screening for food safety. While chromatography offers high specificity, it often suffers from high operational costs (requiring expensive instrumentation and organic solvents) and long analysis cycles. In contrast, the CLEIA method provides a balance of high sensitivity (comparable LODs), low reagent cost (estimated at a fraction of chromatographic costs), and rapid throughput. However, there have been no reports on the detection of bongkrekic acid residues in foods using CLEIA.

This study synthesized an immunogen of BKA via the activated ester method, immunized BALB/c mice with it, and prepared monoclonal antibodies (mAbs) against BKA. Working from the principles of chemiluminescence enzyme-linked immunoassays, we optimized a working system, established an indirect competitive CLEIA (ic-CLEIA) method for bongkrekic acid, and applied the assay to the analysis of BKA residue in food. This method provides a convenient tool for rapid BKA screening to ensure safe food production practices.

## 2. Materials and Methods

### 2.1. Materials and Equipment

Bongkrekic acid, N-hydroxy succinimide (NHS), Freund’s complete adjuvant (FCA), hypoxanthine–aminopterin–thymidine (HAT), Freund’s incomplete adjuvant (FIA), and 1-(3-dimethylaminopropyl)-3-ethylcarbodiimide (EDC) were purchased from Sigma–Aldrich (St. Louis, MO, USA). Toxoflavin (TF) was sourced from Aladdin (Shanghai, China). Monensin (MON), diethylmalonate (DEM), citric acid (CA), deoxynivalenol (DON), and fumonisin (FB) were obtained from Macklin (Shanghai, China). Bovine serum albumin (BSA) and ovalbumin (OVA) were sourced from Yuanye (Shanghai, China). Goat anti-mouse horseradish peroxidase–immunoglobulin G (HRP-IgG), single-component 3,3′,5,5′-tetramethylbenzidine (TMB) chromogenic solution and ECL plus ultrasensitive luminescence solution were acquired from Solarbio (Beijing, China).

The gel electrophoresis system was supplied by Tanon (Shanghai, China). The Nao Drop 2000c UV–Vis spectrophotometer and Multiskan FC microplate reader were supplied by Thermo Scientific (Waltham, MA, USA). A SpectraMaxi3x multifunctional microplate reader was supplied by Molecular Devices (Sunnyvale, CA, USA). A UPLC H-Class-TQD MS system was supplied by Waters Corporation (Milford, MA, USA).

### 2.2. Synthesis and Identification of Artificial Antigens of Bongkrekic Acid (BKA-BSA, BKA-OVA)

**Figure 1:**
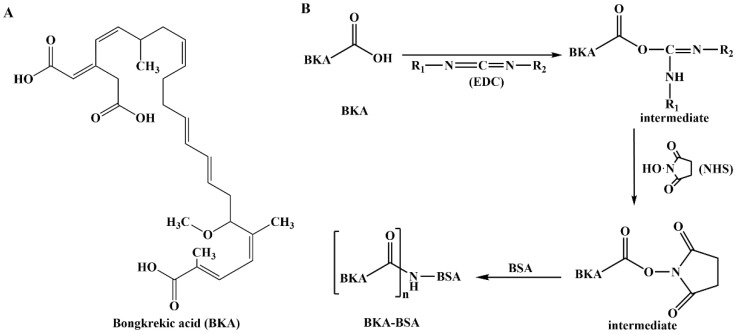
The chemical structural formula of bongkrekic acid (BKA) and synthetic route for bongkrekic acid–bovine serum albumin. (**A**) The chemical strural formula of bongkrekic acid (BKA); (**B**) Synthetic for bongkrekic acid-bovine serum albumin. EDC: 1-ethyl-3-(3-dimethylaminopropyl) carbodiimide; NHS: N-hydroxy succinimide.

Synthesis of BKA-BSA and BKA-OVA was performed via the active ester method (Figure 1). BKA (24.3 mg, 0.05 mmol) was dissolved in 2.5 mL of DMF (solution A), and 7.8 mg (0.05 mmol) of EDC and 5.8 mg (0.05 mmol) of NHS were dissolved in 1.5 mL of phosphate-buffered saline (PBS, pH 7.2) (solution B). Solution A was added to solution B and activated at room temperature in the dark for 6 h (solution C). A total of 33.2 mg (0.0005 mmol) of BSA or 22.5 mg (0.0005 mmol) of OVA was dissolved in 3.0 mL of PBS (solution D). Solution C was slowly added dropwise into Solution D under ice-bath conditions, and the reaction was continued for 8 h. The reaction mixture was subsequently dialyzed and centrifuged to obtain the immunogen of BKA (BKA-BSA). The absorption curves of BKA, BSA and BKA-BSA were scanned at wavelengths of 220–350 nm via a ultraviolet (UV) spectrophotometer, and SDS–PAGE was used to identify the BKA-BSA.

### 2.3. Preparation of Monoclonal Antibodies Against Bongkrekic Acid

All the animal experiments were conducted according to China’s Laboratory Animal Protection Law and Management Law and were approved by the Biomedical Ethics Committee of Zhoukou Normal University, Zhoukou, China (ethics approval number: ZKNU-20240016). The BKA immunogen (BKA-BSA) was mixed with FCA in equal volumes, and after complete emulsification of the mixture, three 7-week-old female BALB/c mice (Laboratory Animal Center of Zhengzhou University) were immunized by multiple subcutaneous injections at the back, with a dose of 95 µg/mouse. Thereafter, the same dose of BKA-BSA was mixed with FIA and emulsified for immunization every 3 weeks, for a total of 5 immunizations. After the immunization procedure was complete, blood was gathered from the tail veins of the mice, and serum was obtained by centrifugation. An enzyme-labeled plate was coated with BKA-coated antigen (BKA-OVA), and the titre of serum antibodies and the inhibitory effect on BKA were analyzed by ELISA [34]. The mice with the highest immune titres and the best BKA inhibitory effects were selected, and shock immunization was performed 3 days before cell fusion (immunization dose of 190 µg). After 3 days, Splenocytes of the mice were prepared, and cell fusion was performed with mouse NS0 myeloma cells. Positive hybridoma cell lines were obtained through HAT selection culture, screening via ELISA, and limiting dilution subcloning, followed by preparation of monoclonal antibodies (mAbs) against BKA by the ascites induction method [35]. The titre and sensitivity of the mAbs were identified.

### 2.4. Indirect Competitive ic-CLEIA Procedure

The detection process used in the ic-CLEIA protocol: (1) Coating: BKA-OVA was used to dilute the designated coating concentration with coating buffer [carbonate buffer solution (CBS)]; 60 μL/well was added to the chemiluminescent enzyme-labeled plate and incubated under optimized coating conditions. The plate was washed 4 times [0.01 mol/L PBST (PBS + 0.5 mL/L Tween-20)] and patted dry with absorbent paper, as described below. (2) Blocking: A 200 μL volume of blocking agent was added to each well and blocked at 37 °C for 60 min, and the plate was washed. (3) Competitive reaction: BKA and anti-BKA mAb (60 μL) diluted in working buffer were added to each well and reacted at 37 °C for 1 h, and the plate was washed. (4) Indirect labeling with reactive antibody: A 60 μL volume of goat anti-mouse HRP-IgG diluted in PBST was added to each well and allowed to react at 37 °C for 1 h, and the plate was washed. (5) Chemiluminescent reaction: A 60 μL volume of luminescent substrate solution was added to each well and incubated in the dark for 5 min. (6) Determination: Relative luminescence unit (RLU) was measured at 425 nm using a SpectraMax i3x microplate reader in luminescence endpoint mode with an integration time of 1000 ms after 5 min of substrate reaction.

### 2.5. Optimization of ic-CLEIA

The coating concentration of BKA-OVA and the dilution of mAbs were optimized by the chessboard array method. BKA-OVA was diluted with the coating solution to concentrations of 1.0, 3.0, 5.0, 7.0, and 9.0 μg/mL and applied horizontally as a coating. The anti-BKA mAb was predefined as five dilutions of 1:500, 1:1000, 1:2000, 1:4000, and 1:8000.

A single-factor optimization test of the ic-CLEIA method was performed as follows. (1) Solutions of 3% casein, 5% skim milk powder, and 1% gelatine were used as blocking solutions. (2) The coating conditions tested were 12 h at 4 °C, 60 min at 37 °C, and 120 min at 37 °C. (3) The competitive reaction times were 20 min, 30 min, 40 min, and 50 min, all at 37 °C. (4) HRP-IgG was diluted at dilutions of 1:2000, 1:4000, 1:8000, and 1:12,000 to measure the concentrations of a series of BKA standards.

All optimization experiments were performed in three independent replicates (n = 3). The above working parameters were determined on the basis of the half-maximal inhibitory concentration (IC_50_) and RLU_max_/IC_50_. The maximum RLU_max_/IC_50_ value and minimum IC_50_ value were considered to indicate the best working conditions.

### 2.6. Sample Pretreatment

Representative high-risk food matrices (tremella, fungus and rice noodles) were selected for recovery investigation, which could fully reflect the practical applicability and anti-matrix interference performance of the pretreatment procedure. An appropriate amount of a representative sample (Tremella, fungus or rice noodles) was taken, crushed or chopped thoroughly, mixed evenly, and put into a clean container as a sample. A total of 3.0 g of the sample was transferred to a 50 mL centrifuge tube; 30 mL of extractant (80% acetonitrile–water solution) was added, and the mixture solution was shaken and mixed thoroughly; 1.2 g of anhydrous sodium sulfate was added; the mixture solution was vortexed for 1 min and centrifuged at 4000 r/min for 4 min; 0.6 mL of the supernatant was taken and air-dried at 60–65 °C; 180 μL of reconstitution solution (pH 8.0, 2 mol/L PBS) was added; and the solution was allowed to rest until detection.

### 2.7. Performance Evaluation of the ic-CLEIA

Under the optimal conditions, with the BKA mass concentration (1.0, 2.0, 4.0, 8.0, 16.0, 32.0, 64.0, 128.0, and 256.0 pg/mL) as the abscissa and the RLU/RLU_0_ as the ordinate (RLU is the relative luminescence value when BKA is added, and RLU_0_ is the relative luminescence value when no BKA is added, that is, RLU_max_), GraphPad Prism 8 software was used for four-parameter fitting to draw the standard curve, and the LOD (IC_10,_ 10% inhibition), limit of quantification (LOQ) (IC_20_, 20% inhibition), IC_50_, and linear range (IC_20_~IC_80_) of BKA were calculated. The inhibition rate formula is as follows: inhibition rate (%) = (1 − B/B_0_) × 100% (B and B_0_ represent positive and negative values).

Three samples (Tremella, fungus and rice noodles) with different BKA contents (40, 100, and 500 ng/kg) were selected; the chemiluminescence values were determined using the ci-CLEIA optimized in this experiment; and the coefficient of variation (CV) was analyzed and calculated. Three different batches of chemiluminescence plates were used to detect the food samples; the chemiluminescence values were read, and the CV was analyzed and calculated.

The cross-reactivity rate (CR%) was used to evaluate the specificity. The larger the CR is, the worse the specificity. The IC_50_ values of BKA analogs (monensin, fumonisin, deoxynivalenol, toxoflavin, citric acid, and diethylmalonate) against BKA monoclonal antibodies were obtained, and the corresponding formula of CR%: CR% = IC_50_ (BKA)/IC_50_ (analog) × 100%.

### 2.8. Verification of the Accuracy of ci-CLEIA

The samples were added at concentrations of 600, 1200, 2400, 4800, and 9600 ng/kg. The differences in the detection results of the two methods were compared. The instrument conditions for UPLC-MS/MS were as follows: chromatographic column: BEH C18 column (100 mm × 2.1 mm × 1.7 μm); flow rate: 0.3 mL/min; column temperature: 40 °C; injection volume: 5 μL; mobile phase: 1% formic acid aqueous solution + acetonitrile. The mass spectrometry conditions: ionization mode: electrospray, negative ion mode; monitoring mode: multiple reaction monitoring (MRM); curtain gas: 0.276 MPa; collision gas: 0.062 MPa; ion spray voltage: 4500 V; ion source temperature: 550 °C.

### 2.9. Date Processing

Excel 2019, GraphPad Prism 8, and Chem Draw Professional 22 were used for tables, inhibition curve, calibration curve, and chemical structures, respectively.

## 3. Results and Discussion

### 3.1. Identification of Complete Antigens and Polyclonal Antiserum Against BKA

**Figure 2:**
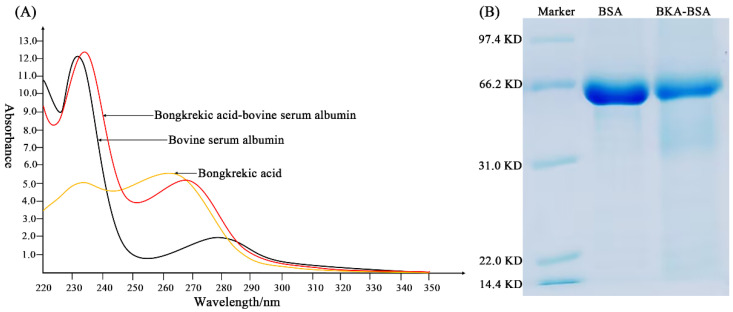
Characterization of the artificial antigen form of bongkrekic acid (BKA). (**A**) Ultraviolet spectra of BKA, bovine serum albumin (BSA) and BKA-BSA. (**B**) SDS–PAGE of a protein marker, BSA and BKA-BSA.

The spectra obtained by scanning BKA, BSA, and BKA-BSA with a UV spectrophotometer (Figure 2A) revealed that the maximum UV wavelengths of BKA, BKA-BSA, and BSA were 267, 269, and 280 nm, respectively. The BKA-BSA was quantified using UV spectrophotometry. The concentration was determined to be 5.14 mg/mL. The absorption peak position of BKA-BSA was offset compared with that of the carrier protein (BSA), and the intensity also changed to a certain extent. Therefore, it can be inferred that the BKA hapten was conjugated to BSA. The gel electrophoresis results for BSA and BKA-BSA (Figure 2B) showed that the migration rate of the BKA-BSA electrophoresis band was slower than that of BSA. This finding indicated that the relative molecular mass of the synthesized artificial antigen was greater than that of the carrier protein, further indicating that BKA was successfully linked to the protein carrier.

BALB/c inbred mice were chosen due to their genetic homogeneity, which reduces experimental variability, and because they are the standard background for hybridoma generation, ensuring compatibility with NS0 myeloma cells for subsequent fusion. The immunization protocol was optimized to elicit a robust humoral response. Mice were initially injected subcutaneously with BKA-BSA emulsified in FCA to prime the immune system, followed by using FIA to enhance polyclonal antibody titer without excessive inflammation. After five rounds of immunizations, the serum titers of the three mice were above 1:12,800, 1:25,600, and 1:25,600, as measured by indirect ELISA (Table 2), indicating that the immunogen (BKA-BSA) has good immunogenicity. The results of indirect competitive ELISA to evaluate the sensitivity of immunized mouse serum showed that the sera of all three mice were sensitive to BKA, with IC_50_ values of 15.4, 5.6, and 7.8 ng/mL (Table 2). This finding strongly confirmed that BKA was correctly coupled to the carrier protein and that the antigenic epitope of BKA could specifically bind to the polyclonal antiserum of mice. After the results of the serum titre and sensitivity analyses, mouse No. 2 was finally selected as the standby mouse for cell fusion.

### 3.2. Performance Evaluation of mAbs Against BKA

Three positive cell lines that could stably secrete anti-BKA mAbs were yielded via cell fusion, hybridoma screening and subcloning; these cell lines were designated 1G4, 2F9, and 3D6. Antibodies were prepared in batches via the in vivo ascites induction method. The titers of BKA mAbs used for indirect ELISA were 1:25,600, 1:512,000, and 1:512,000. The IC_50_ values of mAbs against BKA measured by indirect competitive ELISA were 0.34, 0.26, and 0.23 ng/mL. The subtype of the 3D6 mAb was identified as IgG1 via a mouse mAb subtype identification kit.

### 3.3. Optimization of the ic-CLEIA Working System

**Figure 3:**
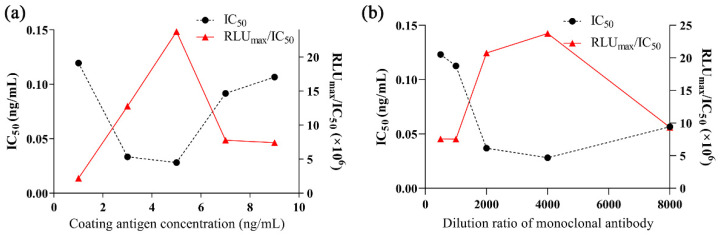
Optimization of the coating concentration of bongkrekic acid–ovalbumin (**a**) and the dilution factor of the anti-bongkrekic acid monoclonal antibody (**b**).

The coating concentration of BKA-OVA and the dilution concentration of the anti-BKA mAb were initially selected by the chessboard method; then, coating source concentrations of 1.0, 3.0, 5.0, 7.0, and 9.0 μg/mL were selected within the upper and lower ranges, and the concentrations of the anti-BKA mAb were set to 1:500, 1:1000, 1:2000, 1:4000, and 1:8000 for competitive reactions. When the coating concentration of BKA-OVA was 5.0 μg/mL (Figure 3a) and the dilution ratio of the anti-BKA mAb was 1:4000 (Figure 3b), the IC_50_ value was at its lowest and the RLU_max_/IC_50_ value was at its highest, indicating that the competitive reaction was most sensitive at this concentration of BKA-OVA and dilution ratio of the anti-BKA mAb; accordingly, a coating concentration of 5.0 μg/mL and a dilution ratio of 1:4000 were selected as the optimal combination.

**Figure 4:**
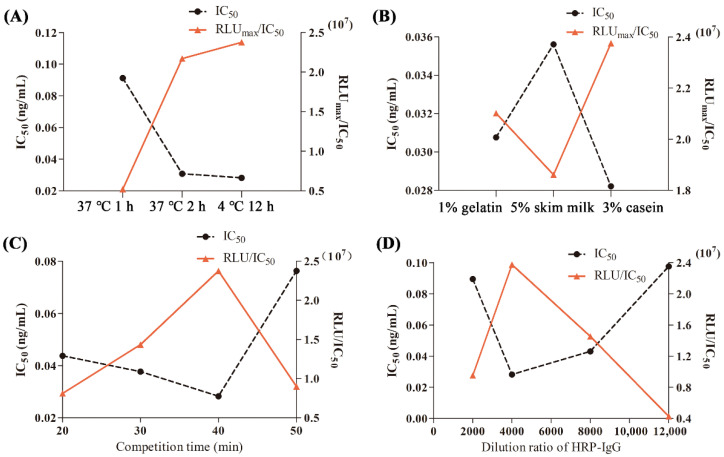
Optimization of working conditions for the indirect competitive chemiluminescence enzyme-linked immunoassay. (**A**) Coating times; (**B**) blocking solutions; (**C**) competitive reaction time; (**D**) dilution ratio of HRP-IgG.

The results of the four sets of single-factor optimization showed that when the coating conditions were 4 °C for 12 h (Figure 4A), the blocking solution was 3% casein (Figure 4B), the competition time was 40 min (Figure 4C), and the dilution ratio of HRP-IgG was 1:4000 (Figure 4D), the corresponding IC_50_ of ci-CLEIA was at its smallest, and the RLU_max_/IC_50_ value was at its largest. The above four conditions were determined to be the optimal working conditions for this method.

There was no significant difference in the blocking effects of the three blocking solutions for the chemiluminescent immunoassay. Casein is a macromolecular protein that can be non-specifically adsorbed on the surface of solid-phase carriers (ELISA plates or chemiluminescent plates), effectively blocking the remaining binding sites on these carriers that are not occupied by antibodies or antigens and reducing nonspecific adsorption and background signals, thereby improving the specificity and sensitivity of detection. Casein also has the advantages of good biocompatibility, high stability, low toxicity, and low cost. The RLU of casein (6.70 × 10^5^) is significantly higher than that of gelatin (6.46 × 10^5^) and skim milk (6.63 × 10^5^). Furthermore, this blocking agent demonstrates superior detection performance, with a lower IC_50_ value and a higher RLU_max_ value, indicating that its signal-to-noise ratio and sensitivity are superior to those of gelatin and skim milk. It can ensure the normal progress of immune reactions and chemiluminescent reactions and is suitable for large-scale experiments and onsite detection.

### 3.4. Analytical Performance of the ic-CLEIA Methods

**Figure 5:**
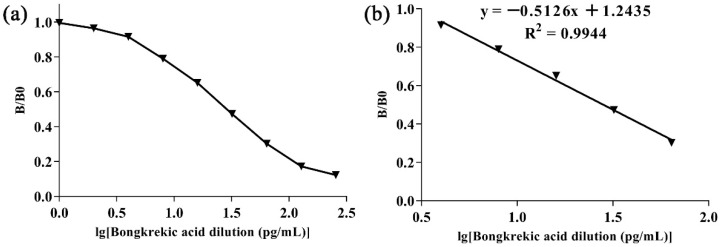
Indirect competitive chemiluminescence enzyme-linked immunoassay (ic-CLEIA) curve for bongkrekic acid. (**a**) Standard inhibition curve of bongkrekic acid; (**b**) calibration curve of bongkrekic acid (4~64 pg/mL).

A standard curve was established with Lg [concentration (BKA)] as the horizontal axis and B/B_0_ as the vertical axis by performing ic-CLEIA detection on BKA with different concentrations of gradient dilution (Figure 5a). It was found that Lg [concentration (BKA)] and B/B_0_ were linearly related, with an equation of y = −0.5126x + 1.2435, R^2^ = 0.9944 (Figure 5b); the IC_50_ was 28.2 pg/mL, the LOD was 4.7 pg/mL, and the detection range (IC_20_~IC_80_) was 7.3~106.6 pg/mL.

Compared with the recently developed TRFMICTS and various immunosensors, the proposed ic-CLEIA method exhibits distinct advantages. Firstly, in terms of sensitivity, the chemiluminescence system provides a lower background and higher signal-to-noise ratio, resulting in an LOD that is 60-fold, 255-fold and 1500-fold lower than those of the TRFMICTS, GICA, and immunosensor methods, respectively (Table 1). Secondly, while immunochromatographic tests are rapid, they are generally semi-quantitative and lack the high-throughput capacity required for large sample volumes [36]. In contrast, our ic-CLEIA method, utilizing a 96-well plate format, enables the simultaneous quantification of dozens of samples, fulfilling the criteria for high-throughput screening. Lastly, although mass spectrometry offers high accuracy, the high cost of equipment and maintenance limits its widespread use; our method strikes an optimal balance between performance, cost, and operational simplicity.

The significantly lower IC_50_ of the developed ic-CLEIA compared with conventional ic-ELISA reported by Wu et al. is mainly attributed to the intrinsic advantages of the chemiluminescence detection principle. Firstly, the chemiluminescence signal has an extremely low background and a higher signal-to-noise ratio than colorimetric ELISA, which greatly improves detection sensitivity. Secondly, the optimized HRP-luminol chemiluminescence system and strictly screened reaction conditions further amplified the effective signal response of the antigen–antibody binding. Thirdly, advanced instrument photoelectric sensing detection (SpectraMax i3x) enables ultra-trace signal capture, while traditional ELISA relies on absorbance readings with relatively weak sensitivity limitations. Therefore, the ic-CLEIA platform realized a remarkable sensitivity improvement, resulting in an approximately 200-fold decrease in IC_50_.

The recovery rates of the spiked concentration of the BKA in a complex mixed matrix (tremella, fungus, and rice noodles) standard were 86.6~94.1%, and the intra-assay and interassay CVs of the ic-CLEIA were 3.6~6.9% and 4.3~8.8%, respectively (Table 3). Both the intra-assay and interassay CVs were lower than 10%, and the interassay CV was greater than the intra-assay CV. Therefore, the established ic-CLEIA for detecting BKA residues had good repeatability.

The IC_50_ values of analogs of BKA [monensin (MON), fumonisin (FB), deoxynivalenol (DON), toxoflavin (TF), citric acid (CA), and diethylmalonate (DEM)] were calculated via the ic-CLEIA method, and the sensitivity of the method to the six competitive analogs was obtained. The cross-reactivity rates of BKA with the six competitive analogs were all less than 0.1% (Table 4), indicating that there was no cross-reactivity with these six analogs, further indicating that the anti-BKA mAb had good specificity.

### 3.5. Comparison of Instrumental Methods

The results of the ic-CLEIA and UPLC-MS/MS methods were compared by statistical analysis (*t*-test) (Table 5). There was no significant difference between the results of the two methods (*p* > 0.05), which further demonstrated that the established ic-CLEIA method for BKA was credible. Bland–Altman analysis revealed a significant correlation between the difference and the mean value (r = 0.997, *p* < 0.001). All points were within the 95% limits of agreement, indicating good consistency between ic-CLEIA and UPLC-MS/MS.

## 4. Conclusions

In this study, an ic-CLEIA method for measuring BKA residues in food was established on the basis of the horseradish peroxidase–luminol chemiluminescent system. After optimization of reaction conditions, the IC_50_ of the ic-CLEIA method was 28.2 pg/mL, the linear range was 7.3~106.6 pg/mL, the recovery rate of addition was 86.6~94.1%, the CVs were both less than 10.0%, and there was no obvious cross-reactivity to other competing analogs. However, potential cross-reactivity with certain structural analogs that were not included in this study cannot be entirely excluded and warrants further investigation. There was no significant difference between the ic-CLEIA results for the food samples and those of the instrumental component analysis (UPLC-MS/MS). The ic-CLEIA method has good sensitivity, precision, accuracy, specificity and stability; simple sample pretreatment requirements; and a detection time of 60~90 min. This method is highly suitable for rapid screening to detect BKA residues in food. In particular, ic-CLEIA is of great significance for research on screening protocols to detect BKA residues in commercially available foods and screening and monitoring work carried out by enterprises, government regulatory departments, etc.

In terms of practical application, the cost–benefit ratio of the proposed method is favorable. The estimated cost per assay is relatively low (approximately $1.0–$2.0), primarily due to the use of conventional reagents. Furthermore, the reliance on a standard microplate reader enhances its applicability in routine testing environments where such equipment is already available. Regarding commercialization, the robustness and stability of the immunoassay components suggest that the development of a commercial kit is feasible. Such a kit could streamline the detection process for operators in food safety or environmental monitoring sectors, reducing variability and training requirements.

## Figures and Tables

**Table 1:** Immunoassays for the determination of bongkrekic acid.

Immunoassays	IC_50_ (ng/mL)	LOD (ng/mL)	Linear Range (ng/mL)	Related References
Ic-ELISA	5.49	0.99	2.12~14.21	Wu et al. (2024) [28]
NLFIA	-	0.66	0~50	Xuan et al. (2024) [31]
TRFMICTS	0.264	0.282	0.1~10	Lin et al. (2024) [29]
GICA	3.6	1.2	1.8~7.2	Cao et al. (2023) [30]
Dual-modular immunosensor	17.9	5.7 (FA), 8.4 (CA)	-	Cao & Li et al. (2023) [32]
Ic-CLEIA	0.028	0.0047	0.0073~0.1066	This study

Ic-ELISA: indirect competitive enzyme-linked immunosorbent assay; IC_50_: half inhibitory concentration; LOD: limit of detection; NLFIA: nanoenzyme-based lateral flow immunoassay; TRFMICTS: time-resolved fluorescent microsphere immunochromatographic test strip; GICA: colloidal gold immunochromatography assay; FA: fluorescence assay; CA: colorimetric assay; Ic-CLEIA: indirect competitive chemiluminescence enzyme-linked immunoassay.

**Table 2:** Detection of the titer and sensitivity of polyclonal antiserum against bongkrekic acid from immunized mice.

Dilution Multiple	Titer of Polyclonal Antiserum Against Bongkrekic Acid		
	No. 1	No. 2	No. 3
2.0 × 10^2^	2.514	2.938	2.736
4.0 × 10^2^	2.153	2.557	2.418
8.0 × 10^2^	1.496	1.840	1.734
1.6 × 10^3^	0.997	1.271	1.186
3.2 × 10^3^	0.556	0.840	0.772
6.4 × 10^3^	0.292	0.576	0.493
1.28 × 10^4^	0.214	0.311	0.265
2.56 × 10^4^	0.142	0.204	0.193
5.12 × 10^4^	0.098	0.136	0.115
Positive control	0.074	0.080	0.077
Blank control	0.059	0.067	0.061
IC_50_ (ng/mL)	15.4	5.6	7.8

**Table 3:** Indirect competitive chemiluminescence enzyme-linked immunoassay for the detection of bongkrekic acid in foods via a recovery test.

Spiked Bongkrekic Acid (ng/kg)	Intraassay			Interassay		
	Mean ± SD (ng/kg)	Recovery (%)	CV (%)	Mean ± SD (ng/kg)	Recovery (%)	CV (%)
40	34.64 ± 2.39	86.6 ± 6.0	6.9	32.69 ± 2.9	81.7 ± 7.3	8.8
100	89.12 ± 4.88	89.1 ± 4.9	5.5	87.26 ± 5.25	87.3 ± 5.3	6.0
500	470.33 ± 16.72	94.1 ± 3.3	3.6	460.37 ± 19.86	92.1 ± 4.0	4.3

**Table 4:** Determination of cross-reactivity to analogs of bongkrekic acid by indirect competitive chemiluminescence enzyme-linked immunoassay.

Competitive Analogs	Chemical Structure	IC_50_	CR%
Bongkrekic acid	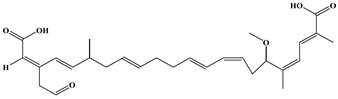		100%
Monensin	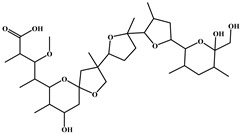	>1.0 × 10^3^	<0.01
fumonisin	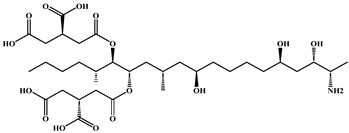	>1.0 × 10^3^	<0.01
deoxynivalenol	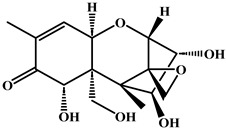	>1.0 × 10^3^	<0.01
Toxoflavin	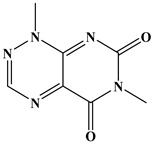	>1.0 × 10^3^	<0.01
citric acid	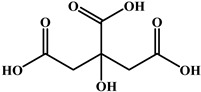	>1.0 × 10^3^	<0.01
diethylmalonate	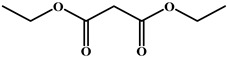	>1.0 × 10^3^	<0.01

**Table 5:** Comparison between indirect competitive chemiluminescence enzyme-linked immunoassay and UPLC-MS/MS.

Spiked Bongkrekic Acid (ng/kg)	ic-CLEIA (ng/kg)	CV (%)	UPLC-MS/MS (ng/kg)	CV (%)
600	563.86 ± 22.91 *^a^*	4.1	566.85 ± 20.36 *^a^*	3.6
1200	1131.33 ± 42.41 *^a^*	3.7	1142.54 ± 36.79 *^a^*	3.2
2400	2275.04 ± 76.15 *^a^*	3.3	2306.49 ± 66.01 *^a^*	2.9
4800	4565.31 ± 166.28 *^a^*	3.6	4666.77 ± 124.28 *^a^*	2.7
9600	9177.84 ± 245.0 *^a^*	2.7	9375.51 ± 289.42 *^a^*	3.1

*^a^* in the upper right indicates no statistical significance between the ic-CLEIA and UPLC-MS/MS (*p* > 0.05).

## Data Availability

The original contributions presented in this study are included in the article/Supplementary Material. Further inquiries can be directed to the corresponding author.

## Supplementary Materials

The following supporting information can be downloaded at: https://www.mdpi.com/article/10.3390/foods15101749/s1, Table S1: Titres of polyclonal antiserum against bongkrekic acid; Table S2: Determination of sensitivity of polyclonal antiserum against bongkrekic acid; Table S3: The indirect ELISA titre of monoclonal antibodies against bongkrekic acid; Table S4: Inhibitive titres and inhibition curves of monoclonal antibodies against bongkrekic acid; Table S5: Optimization of the working concentration of coating antigen; Table S6: Optimization of the working concentration of monoclonal antibody; Table S7: Optimization of four single-factor working conditions of ic-CLEIA; Table S8: Recovery of the indirect competitive CLEIA for bongkrekic acid spiked in food samples; Table S9: Comparison of the indirect competitive CLEIA with HPLC of bongkrekic acid residues in food samples; Table S10: Bland-Altman analysis between ic-CLEIA and UPLC-MSMS; Figure S1: Bland-Altman analysis between ic-CLEIA and UPLC-MSMS.
