# Supplementary figures and images for: Development of a High-Throughput Indirect Competitive Chemiluminescence Enzyme-Linked Immunoassay for the Rapid Detection of Bongkrekic Acid in Tremella Fungus and Rice Noodles
Source: Foods. 2026 May 15;15(10):1749. doi: 10.3390/foods15101749 (PMC13205949; doi:10.3390/foods15101749)

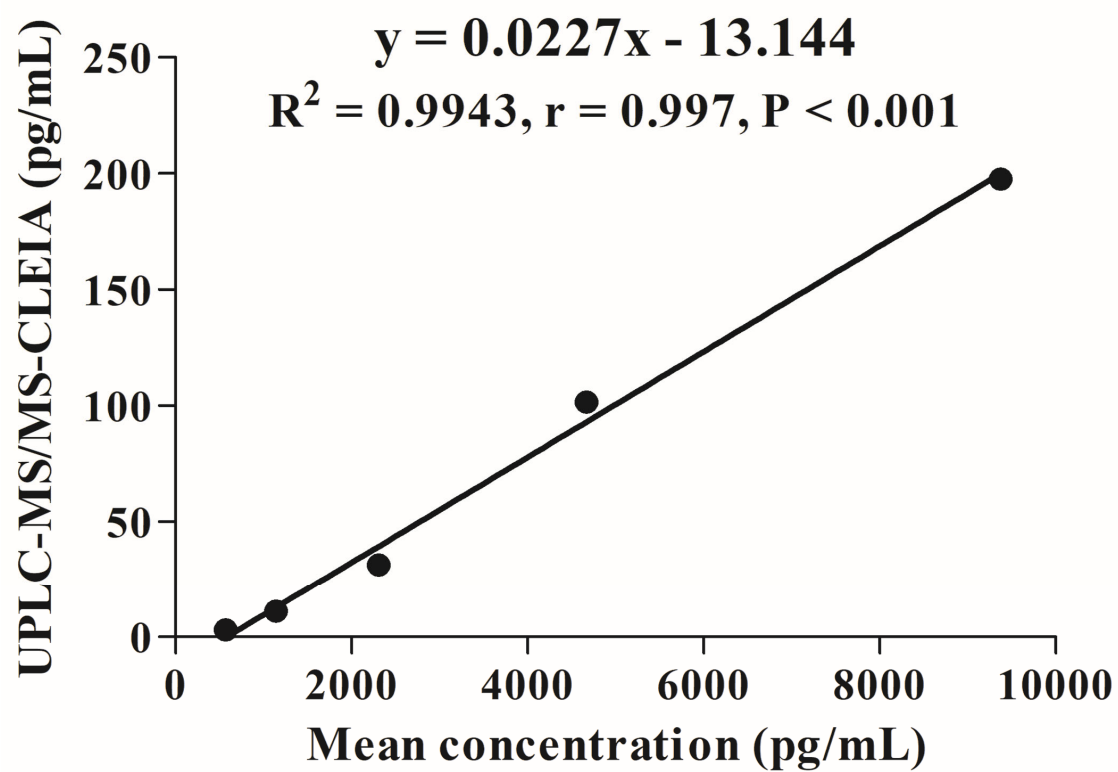

Figure S1. Bland-Altman analysis between ic-CLEIA and UPLC-MSMS.

Supplement: Supplementary file 1 [file foods-15-01749-s001.zip › Figure S1. Bland-Altman analysis between ic-CLEIA and UPLC-MSMS.pdf]
